# Draft Genome Sequence of *Levilinea saccharolytica* KIBI-1, a Member of the *Chloroflexi* Class *Anaerolineae*

**DOI:** 10.1128/genomeA.01357-15

**Published:** 2015-11-19

**Authors:** James Hemp, Lewis M. Ward, Laura A. Pace, Woodward W. Fischer

**Affiliations:** a Division of Geological and Planetary Sciences, California Institute of Technology, Pasadena, California, USA; b Department of Medicine, University of California San Diego, La Jolla, California, USA

## Abstract

We report the draft genome sequence of *Levilinea saccharolytica* KIBI-1*,* a facultative anaerobic member of the *Chloroflexi* class *Anaerolineae*. While *L. saccharolytica* was characterized as an obligate anaerobe, genome analysis provides evidence for the presence of both aerobic respiration and partial denitrification pathways.

## GENOME ANNOUNCEMENT

*Levilinea saccharolytica* KIBI-1 was isolated from sludge granules of a mesophilic wastewater reactor (1). A closely related strain was detected in a trichlorobenzene-transforming microbial consortium (2). *L. saccharolytica* was characterized as an obligately anaerobic, nonmotile, filamentous microbe capable of growth on a range of carbohydrates when supplemented with yeast extract (1). It grows optimally at 37°C and pH 7.0.

The genome of *Levilinea saccharolytica* KIBI-1 (DSM 16555) was sequenced as part of a project to expand the phylogenetic breadth of *Chloroflexi* genomes. Genome sequencing was performed at Seqmatic using the Illumina MiSeq sequencing platform. SPAdes version 3.1.1 (3) was used to assemble the genome. The genome was screened for contaminants based on sequence coverage, GC composition, and BLAST hits of conserved single-copy genes. Genome annotation was performed using the NCBI Prokaryotic Genome Annotation Pipeline. The draft genome is 4.30 Mb in size, assembled into 65 contigs. It encodes 3,672 genes, 3,173 coding sequences, 1 16S RNA, and 46 tRNAs. It is estimated to be ~99% complete based on conserved single-copy genes (110/111).

The *Anaerolineae* described to date have all been classified as strict anaerobes; however, *L. saccharolytica* encodes for a branched aerobic respiration pathway. It has a Complex I (NADH dehydrogenase), Complex II (succinate dehydrogenase), an Alternative Complex III (ACIII) (4), and both an A-family heme-copper oxygen reductase and a *bd* oxidase. It also encodes for two nitrite reduction pathways: a NirS nitrite reductase that reduces nitrite into nitric oxide, and an NrfA protein that reduces it into ammonia. The presence of aerobic respiration genes in *L. saccharolytica* and other recently sequenced *Anaerolineae* suggests that this *Chloroflexi* class is substantially more physiologically diverse than previously recognized (5).

### Nucleotide sequence accession number.

This whole-genome shotgun project has been deposited in DDBJ/EMBL/GenBank under the accession number LGCM00000000.
